# *PAK3* pathogenic variant associated with sleep‐related hypermotor epilepsy in a family with parental mosaicism

**DOI:** 10.1002/epi4.13124

**Published:** 2025-01-13

**Authors:** Antonio Gambardella, Yu‐Chi Liu, Mark F. Bennett, Timothy E. Green, John A. Damiano, Francesco Fortunato, Matthew J. Coleman, Jacqueline Cherfils, Jean‐Vianney Barnier, Jozef Gecz, Melanie Bahlo, Samuel F. Berkovic, Michael S. Hildebrand

**Affiliations:** ^1^ Institute of Neurology, Department of Medical and Surgical Sciences Magna Graecia University Catanzaro Italy; ^2^ Epilepsy Research Centre, Department of Medicine The University of Melbourne, Austin Health Heidelberg Victoria Australia; ^3^ Population Health and Immunity Division The Walter and Eliza Hall Institute of Medical Research Parkville Victoria Australia; ^4^ Department of Medical Biology The University of Melbourne Parkville Victoria Australia; ^5^ Neuroscience Group Murdoch Children's Research Institute, Royal Children's Hospital Parkville Victoria Australia; ^6^ Université Paris‐Saclay, Ecole Normale Supérieure Paris‐Saclay CNRS Gif‐sur‐Yvette France; ^7^ Institut des Neurosciences Paris Saclay, Université Paris‐Saclay, CNRS Saclay Paris France; ^8^ Adelaide Medical School The University of Adelaide Adelaide Australia; ^9^ Neurogenetics Research Program South Australian Health and Medical Research Institute Adelaide Australia

**Keywords:** facial dysmorphism, genetic counseling, *PAK3*, parental mosaicism, sleep‐related hypermotor epilepsy

## Abstract

Protein‐activated kinases mediate spine morphogenesis and synaptic plasticity. PAK3 is part of the p21‐activated kinases (PAKs) family of Ras‐signaling serine/threonine kinases. Pathogenic variants in the X‐linked gene *PAK3* have been described in patients with neurodevelopmental syndromes. We analyzed an Italian family with sleep‐related hypermotor epilepsy, intellectual disability, psychiatric and behavioral problems, and dysmorphic facial features. A novel *PAK3* c.342_344del (p.Lys114del) inframe deletion was detected in the family. Protein structure analysis supported deleterious impact of p.Lys114 deletion through loss or partial loss of autoinhibition of PAK3 protein kinase activity. The male proband had drug‐resistant hypermotor seizures and moderate intellectual disability. His brother had drug‐responsive hypermotor seizures and mild intellectual disability. Both brothers were hemizygous and had psychiatric and behavioral problems as well as dysmorphic facial features. Their mother had never had seizures but was shown to be mosaic for the *PAK3* pathogenic variant. She had normal intellect but did have short stature and dysmorphic facial features similar to her sons. This is the first reported association of a *PAK3* pathogenic variant with sleep‐related hypermotor epilepsy. *PAK3* testing should be considered in families with suspected X‐linked sleep‐related hypermotor epilepsy and intellectual disability, including for mosaicism in mildly affected females.

**Plain Language Summary:**

We studied an Italian family with sleep‐related hypermotor epilepsy, intellectual disability, psychiatric and behavioral problems, and dysmorphic facial features. A novel *PAK3* c.342_344del (p.Lys114del) inframe deletion was detected in the family. Protein structure analysis supported deleterious impact of p.Lys114 deletion through loss or partial loss of autoinhibition of PAK3 protein kinase activity. This is the first reported association of a *PAK3* pathogenic variant with sleep‐related hypermotor epilepsy. *PAK3* testing should be considered in families with suspected X‐linked sleep‐related hypermotor epilepsy and intellectual disability, including for mosaicism in mildly affected females.

**Key points**

- A novel PAK3 inframe deletion was detected in a family with X‐linked sleep‐related hypermotor epilepsy and intellectual disability.
- Predicted impact of p.Lys114 deletion through loss or partial loss of autoinhibition of PAK3 protein kinase activity.
- This is the first reported association of a PAK3 pathogenic variant with sleep‐related hypermotor epilepsy.
- PAK3 testing should be considered for X‐linked sleep‐related hypermotor epilepsy and intellectual disability, including for mosaicism.

## 1 INTRODUCTION

Pathogenic variants in *PAK3* (p21 protein‐activated kinase 3, MIM#300142) have been associated with a non‐syndromic form of X‐linked intellectual disability with recurrent craniofacial abnormalities.^1^, ^2^ Only a small number of families have been reported and most individuals do not have epilepsy or seizures (reviewed in^2^). Most affected family members are male and symptomatic female carriers have only rarely been described.^2^ To our knowledge, mosaicism for *PAK3* variation has not been reported.

We describe an Italian family with a female individual without neurodevelopmental disorder who is mosaic for a novel, rare *PAK3* pathogenic variant. This is only the second individual for whom a detailed clinical description is available.^2^ She transmitted the variant to her two hemizygous sons with epilepsy and mild or moderate intellectual disability, psychiatric and behavioral problems. Strikingly, her two sons share similar facial dysmorphic features, which are more prominent in the proband with more severe neurodevelopmental disorder. This study is the first to describe sleep‐related hypermotor epilepsy in *PAK3*‐related disease. We confirm craniofacial abnormalities even in a mosaic female without neurodevelopmental disorder, further delineating the *PAK3*‐related phenotype and informing genetic counseling for her adult affected sons.

## 2 METHODS

### 2.1 Standard protocol approvals, registrations, and patient consents

The Human Research Ethics Committee of Austin Health, Melbourne, Australia, approved this study (Project No. H2007/02961). Written informed consent was obtained from family members.

### 2.2 Sample collection

Venous blood was obtained from six family members and DNA extracted using standard methods.^3^

### 2.3 Exome sequencing

Exome sequencing was performed using 3 μg genomic DNA. Following amplification and barcoding, the libraries were hybridized to biotinylated complementary RNA oligonucleotide baits from the Agilent SureSelect DNA Human All Exon V5 + UTRs kit (Santa Clara, CA). Amplification was conducted prior to sequencing at 50‐fold depth on the Illumina HiSeq 2500 (San Diego, CA).

### 2.4 Exome sequencing analysis

Exome sequencing reads were aligned with Novoalign version 3.02.13 (http://www.novocraft.com/; Novocraft Technologies Sdn Bhd, Selangor, Malaysia) to the human genome assembly with ambiguous SNPs (hg19 dbSNP135‐masked, UCSC Genome Browser). Germline variant detection was performed with GATK HaplotypeCaller version 3.5–0^4^ and variant annotation using vcfanno^5^ and ANNOVAR.^6^ Exome variants were selected according to the following criteria: located in the splicing or coding region of a gene, appear in the gnomAD database^7^ less than 10 times, and the variant type is missense, nonsense, coding indel or canonical splice site. Several different genetic inheritance models were considered: de novo variants shared by both affected brothers (assuming mosaicism in one parent); homozygous recessive, compound heterozygous recessive, X‐linked recessive inheritance or autosomal dominant inheritance from a mildly affected or non‐penetrant unaffected parent.

### 2.5 PCR and sanger sequencing

*PAK3* was PCR amplified using specific primers available upon request. Amplification reactions were cycled using a standard protocol on a Veriti Thermal Cycler (Applied Biosystems, Carlsbad, CA). Bidirectional sequencing was completed with a BigDye™ v3.1 Terminator Cycle Sequencing Kit resolved using a 3730xl DNA Analyzer (Applied Biosystems).

### 2.6 Droplet digital PCR

We designed a custom assay to detect the novel *PAK3* c.342_344del (p.Lys114del) inframe deletion, as we described previously.^8^ Briefly, following droplet generation, samples were amplified on a C1000 Touch thermal cycler and post‐PCR products were read on the QX200 droplet reader (Bio‐Rad).

## 3 RESULTS

### 3.1 Genetic analysis

**FIGURE 1:**
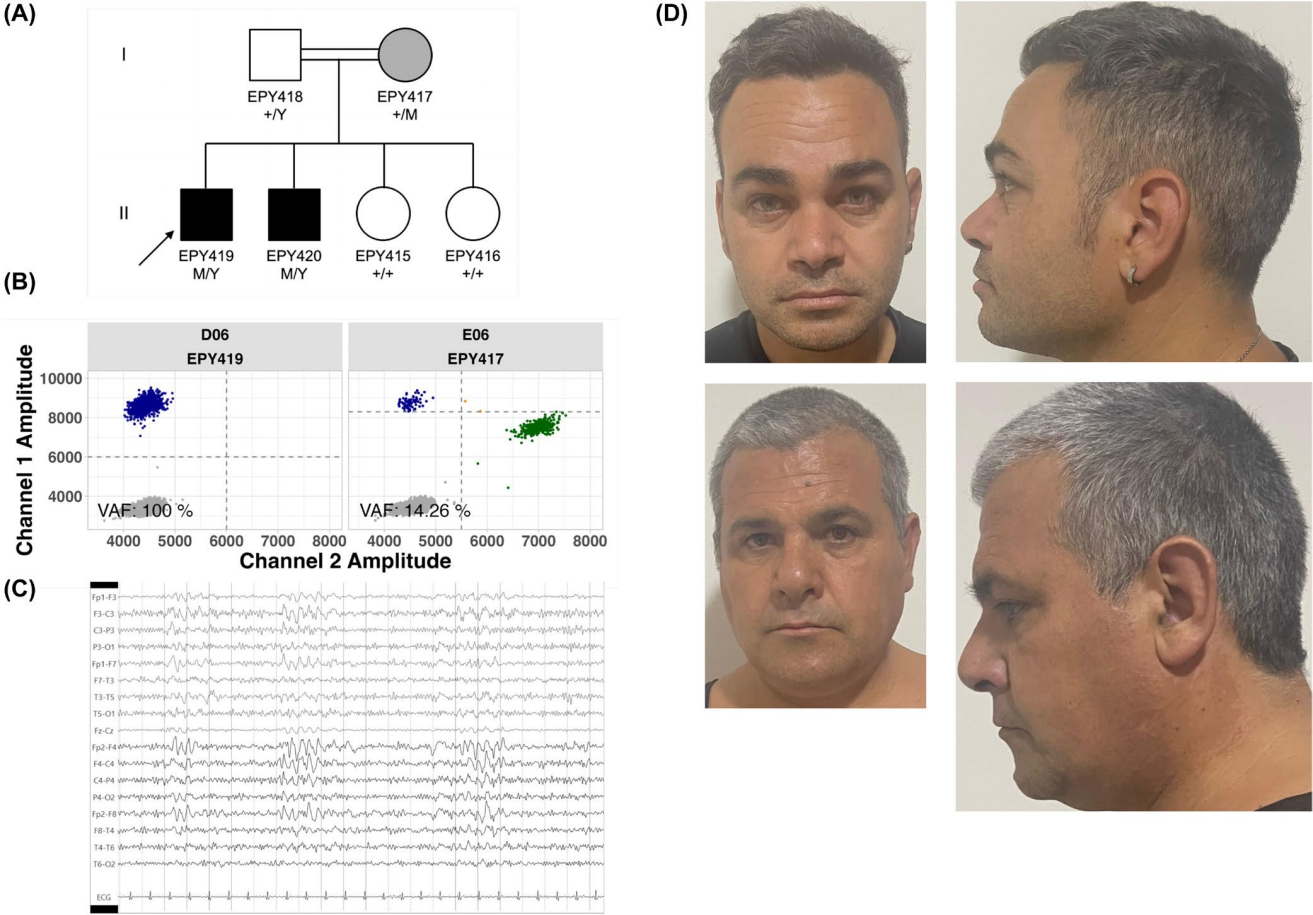
Pedigree, Genotyping and Dysmorphology of the Italian Family. (A) The pedigree of the Italian family showing genotypes based on sequencing and ddPCR analysis. M = *PAK3* c.342_344del (p.Lys114del). Black, affected. Gray mildly affected. White, unaffected. Arrow, proband. (B) The orthogonal validation using droplet digital PCR of the *PAK3* NM_002578.5:C.342_344del, p.(Lys114del) variant in blood‐derived DNA samples of proband (EPY419; II:1) and mother (EPI417; I:2) at VAFs of 100% and 14.26%, respectively. Variant DNA copies (blue droplets), wild‐type DNA copies (green droplets), droplets without DNA copies (gray droplets), droplets with multiple DNA copies (orange droplets). The x‐axis shows the amplitude of wild‐type fluorescent probe; the *y*‐axis shows the amplitude of variant specific fluorescent probe. (C) Electroencephalography (EEG) of the brother (EPY420; II:2) showing bifrontal sharp‐wave discharges. (D) Frontal and lateral views of the proband (EPY419; II:1; Top Panels) showing distinctive facial dysmorphic features including an enlongated face, hypertelorism, a depressed nasal bridge, large ears, a thin upper lip, irregular and large permanent teeth with mild micrognathia. Most of these features were shared by the proband's older brother (EPY420; Bottom Panels) and mother (photo unavailable), although they were more subtle in the mother who was mosaic for the *PAK3* variant.

We detected a novel nonframeshift deletion variant *PAK3* c.342_344del (p.Lys114del) [https://hgvs‐nomenclature.org/stable/] hemizygous in the proband from the Italian family (Figure 1A) via exome sequencing analysis. The p.Lys114del variant was not present in large public databases including gnomAD v4.1.0.^7^ The loss of a positively charged lysine sidechain occurs in the functional GTPase‐binding domain (amino acids 63–123; UniProt: https://www.uniprot.org/uniprotkb/O75914/entry#structure) and autoregulatory region (amino acids 63–150), where other pathogenic variants have been reported.^9^ Segregation analysis by Sanger sequencing revealed the p.Lys114del variant was also present in the proband's affected brother but was not present in his unaffected father or sisters (Figure 1A).

Genomic DNA isolated from venous blood of the proband and his mildly affected mother was analyzed for suspected mosaicism using droplet digital PCR. The *PAK3* p.Lys114del variant was detected at an allele fraction of 100% in the proband as expected given he appeared hemizygous on sequencing, but at only ~14.5% variant allele fraction in the mother, confirming parental mosaicism (Figure 1B).

### 3.2 Protein structure analysis

**FIGURE 2:**
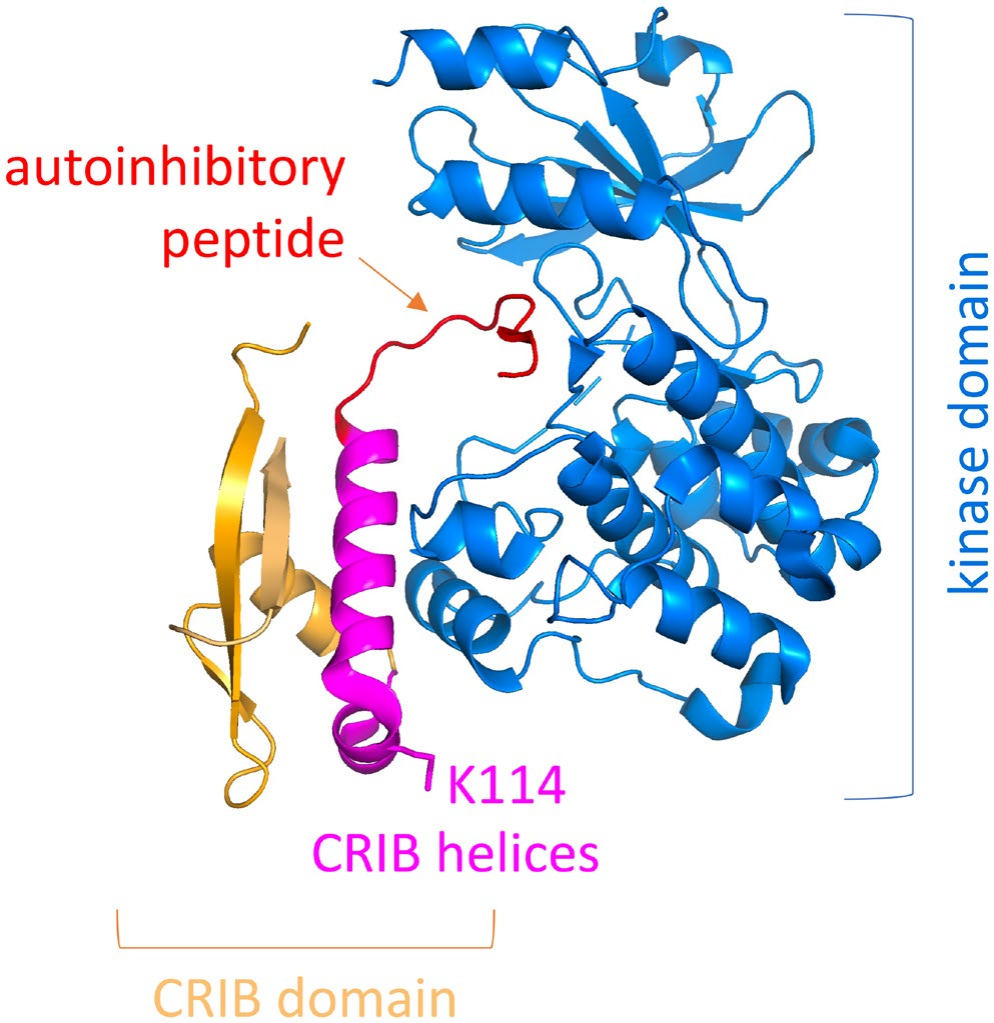
PAK3 p.LYS114 Deletion is Predicted to Disrupt the Structure of the CRIB Domain and Autoinhibitory Lnteractions. The structure is from the autoinhibited PAK1 dimer (PDB 1F3M),^10^ in which the kinase domain is highly similar to the kinase domain of PAK3 (PDB entry 6F3D).^11^ The analysis was carried out with Pymol.

Based on the high sequence and structural similarity of the kinase domain of PAK3 with that of PAK1,^10^, ^11^ Lys114 is predicted to be located in a short loop that connects two alpha helices of the Cdc42‐ and Rac‐interactive binding (CRIB) domain which binds to the kinase domain to position the autoinhibitory peptide into the kinase catalytic site (Figure 2).^10^ Binding of small GTPases is proposed to displace the CRIB domain, resulting in the removal of the autoinhibitory interactions. Deletion of p.Lys114, by making the loop shorter, is predicted to disrupt the structure of the CRIB domain without having an effect on the kinase domain itself, resulting in a weaker CRIB/kinase domain interaction. As a consequence, it should weaken autoinhibitory interactions and increase spontaneous kinase activity, and possibly also facilitate activating interactions with small GTPases at the membrane. The predicted increase in spontaneous and/or GTPase‐stimulated kinase activation would therefore be compatible with a clinical outcome of macrocephaly and intellectual disability or developmental delay and also seizures, as it is observed with known PAK1 variants located in this autoinhibitory domain.^12^, ^13^, ^14^

### 3.3 Phenotypic analysis

The 39‐year‐old male proband had short stature (158 cm; below 3rd percentile, < −2 SD) with borderline microcephaly (occipito‐frontal circumference [OFC]: 54 cm; 3rd percentile, −2 SD), and distinctive facial dysmorphic features including an elongated face, hypertelorism, a depressed nasal bridge, large ears, a thin upper lip, irregular and large permanent teeth with mild micrognathia (Figure 1D). Additionally, he had moderate intellectual disability (IQ: 56) with slurred speech and aggressive behavior which required therapy with olanzapine.

At 4 years of age, he had started to have stereotyped seizures characterized by grunt, vocalization or screaming, eyes opened, head and eyes version, dystonic posturing of his arms (see video recording in Appendix S1). Occasionally, only the initial sequence of these seizures, accompanied by stereotyped screaming, would manifest. These seizures consistently occurred during nocturnal sleep and brief afternoon naps. Interictal EEG recordings revealed bifrontal slow‐sharp waves during wakefulness and non‐REM sleep associated with slow posterior background rhythm. Ictal video‐EEG recordings of habitual seizures disclosed bifrontal spiking activity. Brain magnetic resonance was unremarkable. His electro‐clinical phenotype aligned with criteria for a sleep‐related hypermotor epilepsy (SHE) diagnosis (see video recording in Appendix S1).^15^ He continues to have hyperkinetic motor seizures, almost every two or three months, despite polytherapy with valproate (VPA), oxcarbazepine (OXC), and phenobarbital (PB).

The 46‐year‐old brother displayed normal OFC (57 cm; 50th percentile, 0 SD), subtle dysmorphic facial features such as thin upper lip, large ears, irregular and large permanent teeth with mild micrognathia (Figure 1D). He had borderline intellectual disability (IQ: 70) and exhibited severe irritability with self‐ and hetero‐directed aggressive behavior, necessitating polytherapy with risperidone, olanzapine and promazine. At age of 8 years, he began experiencing sleep stereotyped seizures characterized by eyes opened, facial flushing, grunting meaningless words, head version, hyperkinetic movements consistent with a SHE semiology. Interictal EEG recording revealed bifrontal sharp‐wave discharges during wakefulness and non‐REM sleep associated with a slow posterior background rhythm (Figure 1C). Brain MRI was unremarkable. Monotherapy with carbamazepine had significantly reduced seizure frequency and the addition of PB had rendered him definitively seizure‐free.

The 63‐year‐old mother had short stature (147 cm; below 3rd percentile, < −2 SD) and normal OCF of 54 cm (25th percentile, −0.67 SD). Her intelligence was normal, and she has never had any seizures. She has some dysmorphic facial features including a flat face, mildly down‐slanting palpebral fissures, depressed nasal bridge, broad nasal tip and long ears, and mild micrognathia. The mother declined to be photographed for this study.

## 4 DISCUSSION

Our findings are consistent with a small number of previous studies (reviewed in^2^) reporting *PAK3* pathogenic variants in families with X‐linked neurodevelopmental disorder. Although large deletions have been reported,^2^ this is the first report of a pathogenic nonframeshift deletion and parental mosaicism in *PAK3*‐related disease.

While intellectual disability, psychiatric and behavioral problems, and facial dysmorphism are common and well‐recognized in *PAK3*‐related disease, seizures are less commonly reported, in only 19% of cases.^2^ Sleep‐related hypermotor epilepsy (SHE) is characterized by frequent brief hypermotor seizures in sleep that can look like a simple arousal from sleep, at times confused as a nightmare or night terror [https://www.epilepsy.com/what‐is‐epilepsy/syndromes/sleep‐related‐hypermotor‐epilepsy‐she]. Several genes have been implicated in SHE including nicotinic receptor subunit genes (*CHRNA4, CHRNB2, CHRNA2*), the potassium channel gene *KCNT1*, and GATOR complex genes (*DEPDC5, NPRL2*, *NRPL3*). SHE was diagnosed in the proband and his brother in this study but has not been reported in previous families with *PAK3*, although epilepsy phenotypes have not always been well described (Table 1).^2^, ^16^, ^17^, ^18^, ^19^, ^20^, ^21^, ^22^, ^23^, ^24^, ^25^, ^26^

**TABLE 1:** Reported EEG findings in patients with *PAK3* pathogenic variants.

Study	No. patients	*PAK3* variant	EEG findings
17	4	p.Trp446Ser	Posterior slow waves without epileptic discharges in 4 family members. One family member diagnosed with epilepsy
19	1	p.Ser527Gly	Background dysrhythmia and bifrontal paroxysmal slowing but no epileptiform abnormalities at age of 7. Bilateral central parietal slowing and bilateral mid central parietal sharp waves maximal on the right side at age 12 years. Background dysrhythmia, delta in the right occipital temporal area, and right hemisphere sharp waves maximal in the right posterior temporal region at 14y10mth
20	1	c.276 + 4A > G	Occasional generalized seizures with normal EEG
21	2	p.Lys389Gln	EEG completed in both cousins but not described. Both cousins died at 6 and 8 years of age from cardiorespiratory arrest during a prolonged epileptic seizure
22	2	p.Cys371Tyr	A 24‐h sleep EEG showed little abnormality in the proband at 4 years old. The brother had a normal EEG
23	1	p.Met493Cys	Reported to have epilepsy but no EEG findings
24	1	p.Val326Leu	Normal EEG
25	1	p.Pro193Ser	Normal EEG
26	1	p.Thr131Arg	Reported to have drug‐resistant focal epilepsy but no EEG findings
2	1	p.Lys389Thr	No history of epilepsy. No EEG findings
27	1	p.Ala365Glu	Reported to have myoclonic epilepsy but no EEG findings
28	1	p.Gly424Arg	Reported to have seizures during childhood but no EEG findings

In this family, the affected brothers had onset of hypermotor, versive and focal to bilateral tonic–clonic seizures at 4 (proband) and 7 years, respectively, which were drug‐resistant in the proband, and occurred predominately at night. Almost all reported cases with *PAK3* pathogenic variants have intellectual disability,^2^ consistent with the mild or moderate intellectual disability of the affected brothers reported here. Psychiatric and behavioral disturbances including attention deficit and hyperactivity disorder and autism spectrum disorder have also been frequently observed, in the Italian family or elsewhere.^2^ Although the head circumference of the proband appears borderline microcephalic, both the proband and his mother have short stature meaning relative to height they may in fact have macrocephaly which is commonly associated with *PAK1* pathogenic variants.^12^, ^13^, ^14^ However, reported electro‐clinical features of *PAK1*‐associated neurodevelopmental disorder are distinct from those in *PAK3* neurodevelopmental disorder including multi‐focal spike wave and epileptic discharges, and sharp waves in frontal and temporal regions.^27^

Recent genetic evidence highlight that the Rac/Cdc42 pathway of which PAK3 is part plays a major role in brain development and functions and its mutational dysfunction leads to neurodevelopmental disorder.^28^, ^29^, ^30^, ^31^, ^32^, ^33^ PAK3 is a member of group I of p21‐activated kinases, which are the main effectors of Rac1/Cdc42 GTPases.^34^ These kinases consist of a carboxy‐terminal catalytic domain and an amino‐terminal regulatory region comprising two overlapping domains, a GTPase‐binding domain and an autoinhibitory domain.^34^ They regulate several pathways such as the LimKinase/Cofilin and RAF/MEK/ERK pathways, but also phosphorylate several other targets involved in various biological processes. They also interact with numerous proteins, including Nck adaptor proteins and GEFs of the PIX family.

All three PAKs are involved in brain development, and their variations are responsible for various neurodevelopmental disorders. As PAK3 is located on the X chromosome, most patients are male, and a few hemizygous females with X chromosome inactivation mosaicism have also been described.^2^ Around twenty pathogenic or likely pathogenic *PAK3* variations have been reported in ClinVar [https://www.ncbi.nlm.nih.gov/clinvar/], some with clinical descriptions. These include a wide clinical spectrum ranging from a moderate non‐syndromic form of ID to severe cases of profound ID, with brain abnormalities such as microcephaly, macrocephaly and callosal agenesis, and with several psychiatric traits such as stereotypy, self‐mutilation and epilepsy (Table 1). Most reported variants are missense substitutions localized to the kinase domain.^28^ The p.Lys114del variant reported here is the first indel located in the autoinhibitory domain.

The primary predicted functional effect of the p.Lys114del variant, based on protein structure analysis, is a loss or partial loss of autoinhibition of PAK3 kinase activity, which is known^35^ to lead to a phenotypic outcome of macrocephaly with intellectual disability or developmental delay. Another possible mechanism is through partial loss of homodimerization with PAK1 and thus altered transregulation of PAK3 by PAK1 and/or increased spontaneous kinase activity.^36^ Either way this p.Lys114del variant offers further opportunity to delve deeper into the complex mechanisms of PAK1‐PAK3 transregulation in neurodevelopment. The p.Lys114del variant is located in the CRIB/PBD binding domain where one other pathogenic variant is reported.^9^

Most reported cases with *PAK3* deficiency have been male with their variant inherited from unaffected carrier mothers.^2^ Here we report a rare mildly affected mother with a *PAK3* pathogenic variant. Interestingly the mother, who is mosaic for the deletion, only had facial dysmorphism and short stature, and none of the neurodevelopmental features. Strikingly, the facial dysmorphic features were shared by her sons, albeit being more prominent, particularly in the proband. The phenotype of the mosaic mother may reflect a dominant effect of the p.Lys114del variant leading to constitutive activation of PAK3 kinase activity, an effect that could be compounded by skewed X‐inactivation which has been reported in carrier females with pathogenic germline *PAK3* variants and mild phenotypes.^16^ However, we were unable to obtain fresh blood or other tissues from the mother to determine her X‐inactivation status. Both of her hemizygous sons will transmit the *PAK3* pathogenic variant to any daughters, and given they only have mild or moderate intellectual disability, counseling will be recommended.

## 5 CONCLUSIONS

In cases with suspected X‐linked sleep‐related hypermotor epilepsy, particularly in males with nocturnal seizures, intellectual disability, psychiatric and behavioral problems, and facial dysmorphisms, *PAK3* gene sequencing should be performed in addition to standard clinical microarrays for detection of large deletions at the *PAK3* locus. Mildly affected females should be tested for carrier status or mosaicism. Finding a pathogenic variant in *PAK3* will help clinicians with diagnosis and genetic counseling, with implications for expressivity of both severe (e.g., sleep‐related hypermotor epilepsy, intellectual disability), and milder (e.g., facial dysmorphism) phenotypes, depending on whether the pathogenic variant is inherited by a male or female offspring, or is gonadal mosaic in female offspring.

## CONFLICT OF INTEREST STATEMENT

The authors declare no conflicts of interest. We confirm that we have read the Journal's position on issues involved in ethical publication and affirm that this report is consistent with those guidelines.

## Supporting information


**Appendix S1.** Video showing ictal EEG recording of the proband. Seizure arose out of sleep stage II and the EEG showed a diffuse sharp‐slow wave followed by muscular artifacts with intermixed diffuse delta waves with some bifrontal predominance. During the seizure, the proband roused and sat up from a supine position. Then the proband became agitated and looked around with facial grimacing and dystonic movements of his arms.

## Data Availability

The *PAK3* pathogenic variant has been submitted to LOVD database (https://www.lovd.nl/). Sequencing data are available from the corresponding authors on reasonable request.
