# Supplementary figures and images for: PAK3 pathogenic variant associated with sleep‐related hypermotor epilepsy in a family with parental mosaicism
Source: Epilepsia Open. 2025 Jan 13;10(2):593–601. doi: 10.1002/epi4.13124 (PMC12014923; doi:10.1002/epi4.13124)

**Appendix S1**


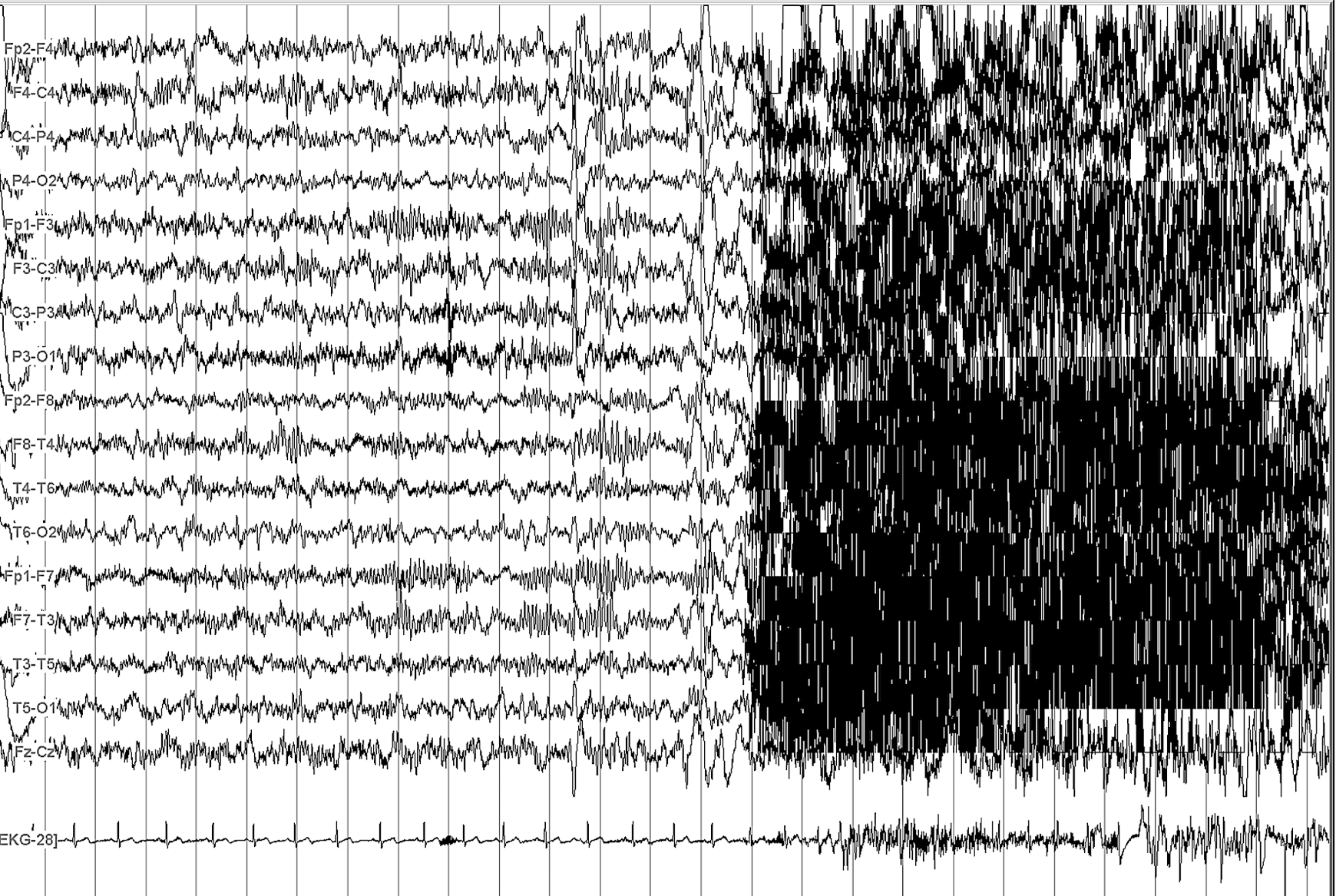


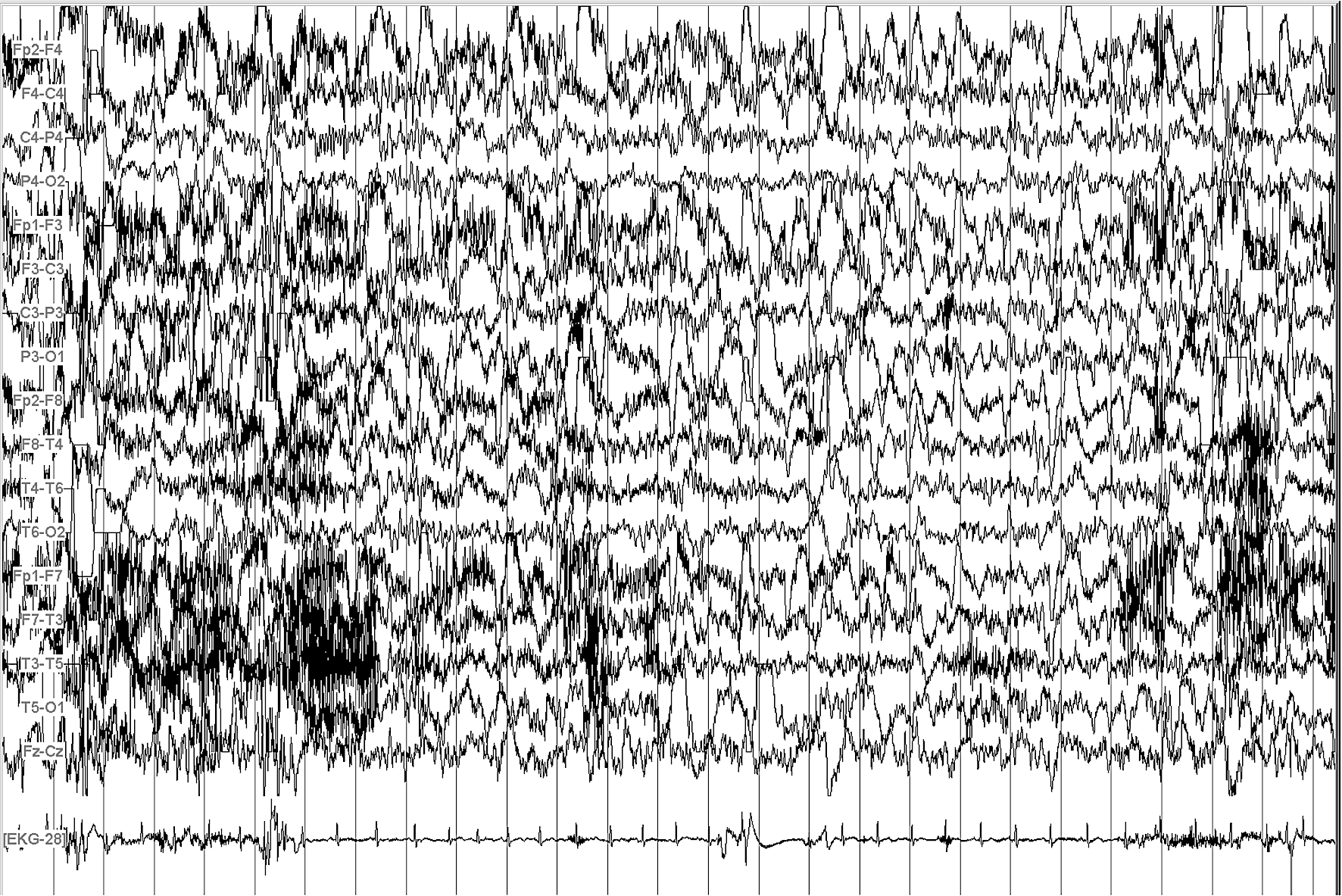

Supplement: Supplementary file 1 — Appendix S1. Video showing ictal EEG recording of the proband. Seizure arose out of sleep stage II and the EEG showed a diffuse sharp‐slow wave followed by muscular artifacts with intermixed diffuse delta waves with some bifrontal predominance. During the seizure, the proband roused and sat up from a supine position. Then the proband became agitated and looked around with facial grimacing and dystonic movements of his arms. [file EPI4-10-593-s001.docx]
